# Decrease in Chitinase 3-Like Protein 1 Levels Reflects Improvement in Liver Fibrosis after HCV Eradication

**DOI:** 10.1155/2020/8539804

**Published:** 2020-10-01

**Authors:** Qian Kang, Jianhong Chen, Hao Luo, Ning Tan, Hui Gao, Xiaxia Zhang, Min Yu, Dan Liu, Hongli Xi, Yaoyu An, Yifan Han, Ran Cheng, Xiaoyuan Xu

**Affiliations:** ^1^Departments of Infectious Diseases, Peking University First Hospital, NO.8, Xishiku Street, Xicheng District, Beijing 100034, China; ^2^Department of Gastroenterology, Capital Medical University Affiliated Beijing Shijitan Hospital, Beijing 100038, China; ^3^Department of Gastroenterology, Capital Medical University Beijing Tiantan Hospital, Beijing 100070, China

## Abstract

**Aim:**

The success of direct-acting antivirals (DAAs) against hepatitis C virus is a major breakthrough in hepatology. Previous studies have shown that chitinase 3-like protein 1 (CHI3L1) was a marker for staging of liver fibrosis caused by HCV. In this investigation, we used CHI3L1 as a surrogate marker to compare dynamic hepatic fibrosis variations following the elimination of HCV among cases receiving sofosbuvir (SOF)-based regimens and pegylated interferon/ribavirin (PR) treatments.

**Methods:**

The study enrolled 105 patients, including 46 SOF-based regimens treated patients, 34 PR-experienced patients, and 25 untreated patients. Serum samples and clinical data were obtained at the baseline, the end of treatment, and at weeks 24 and 48 after treatments.

**Results:**

First, we found that serum level of CHI3L1 correlated moderately but significantly with LSM (*r* = 0.615, *P* < 0.001) at the baseline, and diagnosed liver cirrhosis at baseline with high accuracy (AUC = 0.939) by ROC analysis. So we explored CHI3L1 as a sensitive biomarker to monitor the regression of liver fibrosis after HCV eradication. We found that the serum CHI3L1 level of CHC cases receiving SOF-based regimen treatments was markedly reduced immediately after treatment compared with that at the baseline (123.79 (118.55) vs. 118.20 (103.68), *P* = 0.001). For cases undergoing PR treatment, the serum CHI3L1 decreased significantly at week 24 posttreatment compared with that at the baseline (69.98 (51.44) vs 89.15 (110.59), *P* = 0.016). For the untreated cirrhotic patients, CHI3L1 levels increased at week 96 follow-up compared with that at the baseline (194.73 (172.46) vs. 89.50 (242.97), *P* = 0.048), reflecting continued worsening of liver fibrosis.

**Conclusion:**

CHI3L1 is suggested to be the sensitive marker to monitor fibrosis variations in weeks during treatments and after achieving SVR. It has the potential to allow the identification of early treatment failure for a timely switch to alternative treatment and to allow monitoring progression of fibrosis as a risk factor for liver cirrhosis.

## 1. Introduction

Infection with chronic hepatitis C (CHC) is still a notable burden in the world. As estimated by the World Health Organization (WHO), 71 million people in the world suffer from infection with hepatitis C virus (HCV) in 2015; besides, 399,000 people die of HCV infection-induced hepatocellular carcinoma (HCC) or cirrhosis. In order to cure viral hepatitis by the year of 2030 as a threat to public health [1], the direct-acting antivirals (DAAs) have been developed to achieve high sustained virological response (SVR) rates in HCV treatment compared with the traditional peginterferon and ribavirin (PegIFN/RBV, PR) treatments [2].

Ultimately, HCV elimination aims to avoid liver disease progression. Previous studies have shown that after receiving IFN-based treatment regimens to obtain SVR, CHC patients' liver fibrosis (LF) progression was reversed or halted, resulting in decreased incidence rates of HCC and other liver-related complications, as well as enhanced quality of life related to health [3, 4]. However, there have been reports showing that, for patients with HCV infection-related cirrhosis, HCV infection resolution induced by DAA shows no effect on diminishing HCC occurrence, and those with HCC are still associated with an increased risk of short-term tumor relapse [5, 6].

Liver biopsy is the most reliable approach to diagnose liver fibrosis; nonetheless, its application is restricted due to its invasive nature, and it is not feasible to conduct serial liver biopsies to diagnose and monitor CHC patients after HCV eradication [7]. The noninvasive transient elastography (TE) is the alternative to liver biopsy, which has replaced biopsy in some circumstances [8]. However, FibroScan, with its limited dynamic range and great operator-related variability, is not sensitive enough to determine minor changes in fibrosis, and it is not available in every hospital. Therefore, a more sensitive noninvasive serum biomarker to evaluate liver fibrosis during and after HCV treatments is still an unmet need.

Chitinase 3-like protein 1 (CHI3L1) is a member of the chitinase family and expressed most abundantly in the liver among major human tissues analyzed [9]. CHI3L1 can promote hepatic stellate cell (HSC) activation and proliferation, therefore directly participating in the formation and maintenance of hepatic fibrosis [10]. As suggested in prior research, CHI3L1 may be adopted to diagnose cirrhosis or liver fibrosis caused by viral hepatitis and alcoholic hepatitis, and it could accurately distinguish among different stages of LF [10–13].

In this paper, we explored using the dynamic changes of CHI3L1 levels to be the serum marker to monitor liver fibrosis changes with high sensitivity in a noninvasive manner before and after treatment for CHC patients. At the same time, we assessed the ability of CHI3L1 in determining differences in treatment efficacies of liver fibrosis improvement with the DAA treatment and the PR treatment regimens for CHC patients.

## 2. Materials and Methods

### 2.1. Patients

The current work was performed at the Department of Infectious Diseases in the Peking University First Hospital. Altogether, 105 CHC patients were recruited into this study between January 2015 and December 2016. The study included 46 patients treated with the sofosbuvir (SOF)-based regimens, 34 PR-experienced patients, and 25 untreated patients.

Forty-six patients who participated in the study were those who gained SVR to the SOF-based therapy and satisfied those inclusion criteria: (1) age > 18 years old, male or female; (2) HCV RNA-positive for at least 6 months before baseline, or the liver biopsy conforming to chronic infection with HCV; and (3) HCV genotype 1b or 2a, DAA-naive, and gained SVR to the SOF-based therapy. The following were exclusion criteria: (1) other hepatitis virus/human immunodeficiency virus (HIV) infection; (2) other chronic liver diseases; (3) with HCC and other malignancies; (4) with severe cardiovascular and cerebrovascular diseases; (5) eGFR < 50 ml/min/1.73 m^2^; (6) pregnant or lactating women; (7) treatment failure (nonresponse or relapse) patients; and (8) incomplete clinical information or blood samples not available.

Thirty-four CHC patients received treatments with PR and gained SVR, and twenty-five untreated patients also took part in this work. Other inclusion criteria and exclusion criteria were the same as those mentioned for the SOF-based regimens above.

Each eligible patient had provided informed consent to participate in this study. Our study protocol had gained approval from the Ethics Committee of Peking University People's Hospital (Approval No.19 for the year of 2009) and was based on the ethical guidelines in the Declaration of Helsinki.

### 2.2. Data Collection

Clinical data, HCV RNA quantitation, HCV RNA genotyping, liver stiffness measurement (LSM), and hematological and biochemical tests were performed at the baseline. Clinical data and blood samples were gathered at the baseline (T0), the end of the treatment (T1), and 24-week (T2) and 48-week posttreatments (T3) in cases receiving SOF-based and PR treatments. In addition, blood samples and clinical data were obtained at baseline (U0), at the 24-week follow-up (U1), the 48-week follow-up (U2), the 72-week follow-up (U3), and the 96-week follow-up (U4) in the untreated patients. Blood samples were frozen in a refrigerator at -80°C.

The Virus Laboratory at the Department of Infectious Disease of Peking University First Hospital was responsible for the quantification and genotyping of HCV RNA. The HCV RNA in serum was quantified by the use of COBAS Taqman HCV Assay Kit (Roche Molecular Systems Inc., Pleasanton, CA, USA). Transient elastography (Fibroscan, Echosens, Paris) was adopted to measure LSMs, and LSM ≥ 13 kPa was set as the standard to diagnose cirrhosis [14]. The Clinical Laboratory of Peking University First Hospital was responsible for blood and biochemical tests.

### 2.3. Enzyme-Linked Immunosorbent Assay (ELISA)

The CHI3L1 ELISA kit (Hangzhou Proprium Biotech Co. Ltd., Hangzhou, Zhejiang, China), which was approved by the SFDA and had CE mark, was used to quantify CHI3L1 contents in serum.

### 2.4. Statistical Analysis

Data were collected and analyzed using Microsoft Excel (Microsoft, Redmond, Washington, USA). Those normally distributed continuous variables were presented in the form of means ± standard deviation (SD) and analyzed using one-way analysis of variance (ANOVA) and Student's *t* test. Those variables conforming to nonnormal distribution were expressed in the manner of medians (interquartile ranges) and analyzed using the nonparametric tests, including Kruskal-Wallis test and Wilcoxon matching pair test. Besides, those categorical variables were presented in the manner of absolute percentages and numbers and analyzed through Fisher's exact test and chi-square test. Correlations of CHI3L1 contents in serum with LSM were analyzed through Spearman's rank test. The CHI3L1 performance in the discrimination of patients with liver cirrhosis from those with not was assessed using the receiver operating characteristic (ROC) curve. The SPSS 16.0 software was adopted for all statistical analyses. A difference of *P* < 0.05 indicated statistical significance.

## 3. Results

### 3.1. Baseline Characteristics

Table 1 presents the major virological, demographic, and clinical characteristics. Of the 46 SOF-based regimens treated patients, their mean age was 56.76 ± 14.53 years. For the PR-experienced patients, 34 patients have an average age of 48.12 ± 11.64 years. Of the 25 untreated patients, their mean age was 58.24 ± 16.60 years. Besides, there were significant differences in ages between PR-experienced and DAA-treated or untreated patients; no other baseline characteristics showed significant difference across these 3 groups (Table 1).

### 3.2. Correlation Analysis

**Figure 1:**
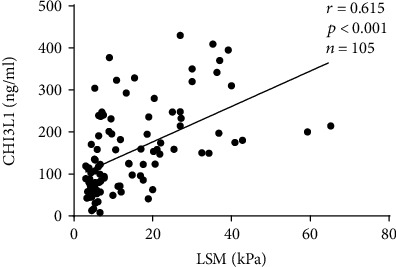
Correlation between serum CHI3L1 levels and LSM at baseline in all patients. There were positive correlations between serum CHI3L1 levels and LSM (*r* = 0.615, *P* < 0.001).

For all patients, we found a moderate correlation between serum CHI3L1 level and LSM at baseline (*r* = 0.615, *P* < 0.001) (Figure 1).

### 3.3. Diagnostic Performance of CHI3L1 for Liver Cirrhosis

**Figure 2:**
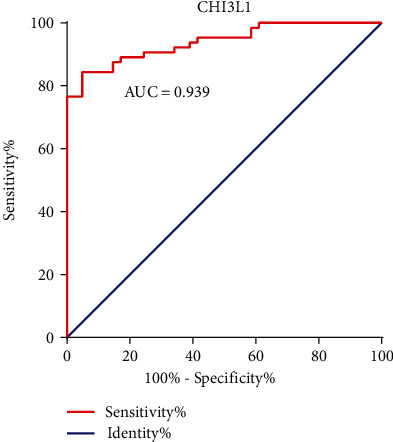
Area under the ROC curve of CHI3L1 in the diagnosis of liver cirrhosis. ROC: receiver operating characteristic curve.

To analyze the predictive value of CHI3L1 for liver cirrhosis patients, we generated a ROC curve. Thirty-nine liver cirrhosis patients and 66 noncirrhotic cases were recruited into this study. The area under the ROC curve value for CHI3L1 to predict liver cirrhosis was 0.939 (95% CI, 0.8974-0.9814), with 95.1% sensitivity and 84.4% specificity at the cutoff point at 141.23 ng/ml (Figure 2).

### 3.4. The Serum CHI3L1 Level Decreased Faster in the DAA-Treated Patients than the PR-Experienced Patients

**Figure 3:**
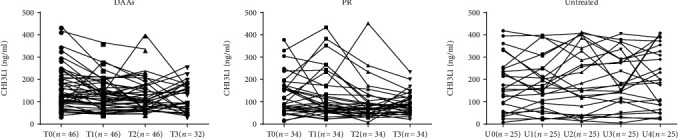
Dynamic changes of serum CHI3L1 in different treatment groups at different time points. T0: baseline; T1: end of treatment; T2: 24 weeks after treatment; T3: 48 weeks after treatment. U0: baseline; U1: follow-up of 24 weeks; U2: follow-up of 48 weeks; U3: follow-up of 72 weeks; U4: follow-up of 96 weeks.

As shown in Figure 3, the serum CHI3L1 levels of the CHC patients treated with SOF-based regimens decreased significantly at the end of treatments compared with those at the baseline (123.79 (118.55) (the median and interquartile range) vs. 118.20 (103.68), *P* = 0.001). The decline continued to week 24 and week 48 after treatment, but at a much slower pace with no statistical significance between the later interval. In the patients treated with PR, the speed of the decrease in the serum CHI3L1 level was slower than that of the DAA-treated patients—it only reached statistical significance at week 24 posttreatment compared with the baseline (89.15 (110.59) vs. 69.98 (51.44), *P* = 0.016). In contrast, for the untreated cirrhotic patients, their CHI3L1 levels increased at the week 96 follow-up compared with those at the baseline (224.18 (267.93) vs. 194.73 (172.46), *P* = 0.048); however, the CHI3L1 level seemed to be stable in the untreated noncirrhotic patients during 96 weeks follow-up. The serum CHI3L1 level decreased faster in the DAA-treated patients than in the PR-experienced patients (Figure 3 and Table 2).

### 3.5. Changes in the CHI3L1 Level Stratified Liver Fibrosis Treatment Responses into Different Categories for Different Treatment Regimens

**Figure 4:**
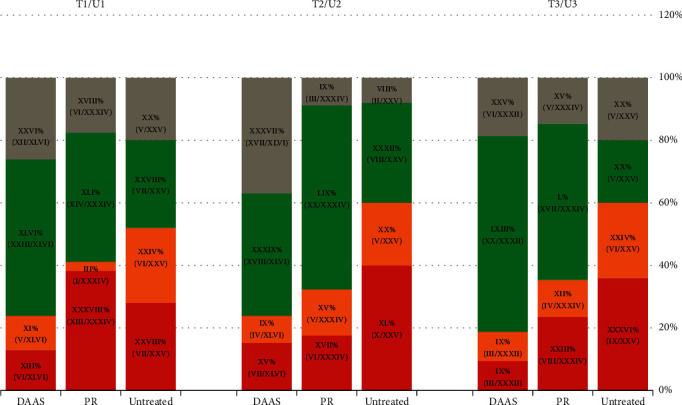
Distributions of different categories of responses to the treatments by CHI3L1. T1/U1: at the end of treatment; T2/U2: at 24 weeks after treatment; T3: at 48 weeks after treatment. Red color indicates rapid ascenders, and yellow color indicates slow ascenders; green color indicates rapid decliner, and grey color indicates slow decliners.

Based on the amount of changes from the baseline to posttreatments, we categorized the treatment responses into four categories: the rapid decliner, the slow decliner, the rapid ascender, and the slow ascender. The rapid decliner referred to a patient with a 20% or more decrease from the baseline level, and the slow decliner referred to a patient with a less than 20% decrease. In parallel, the rapid ascender and slow ascender were defined as a patient with a >20% or <20% increase, respectively. For the untreated group, we found roughly even distributions of patients in the 4 categories (Figure 4).

For the SOF-based regimen-treated patients, 76.1% (35/46) of the patients had a lower serum CHI3L1 level at the end of their treatment than that at the baseline. Nonetheless, 23.9% (11/46) of the patients had a higher CHI3L1 level. In this group, the rapid decliner was the largest category, comprising 50% of the patients given SOF-based treatment.

For the PR-experienced patients, 58.8% (20/34) of the patients had a serum CHI3L1 level lower at the end of treatment than at the baseline. A total of 41.2% (14/34) of the patients had a higher CHI3L1 level. At week 48, only 9.4% of the patients were rapid ascenders in the SOF-based treatment group; however, 23.5% (approximately 2.5 times more) of the PR-based regimens patients were rapid ascenders at the same time point.

### 3.6. Dynamic Changes in the CHI3L1 Levels Reflected Expected Differences in Treatment Responses of Different Treatment Regimens

As we can see from Figure 4, the improvement in fibrosis indicated by the percentage of decliners (adding the percentages of those represented by the green and grey colors) in the DAA-treated group started at 76.1% at the end of treatment, remained at 76.1% at week 24, and rose to 81.2% at week 48. The improvement in fibrosis indicated by the percentage of decliners in the PR-treated regimens patients started at 58.8% at the end of treatment, peaked at 67.7% at week 24, and remained at 64.7% at week 48.

At 48 weeks after treatment, only 18.8% of the patients were ascenders in the DAA-treated group. In contrast, 35.3% of the patients were ascenders in the PR-treated group, and 60% of the patients were ascenders in the untreated group.

In addition, the SOF-based regimens group showed a higher percentage of decliners (76.1%, 38/46 patients) than the PR-based regimens group, which had only 58.8% (20/34) decliners, at the end of treatment, suggesting that the SOF-based treatments more efficiently reduced liver fibrosis than the PR treatment regimens immediately after treatment; in addition, alterations of CHI3L1 content might serve as the sensitive marker to monitor liver fibrosis changes within a short term in a noninvasive way (measured in weeks rather than in months).

**Figure 5:**
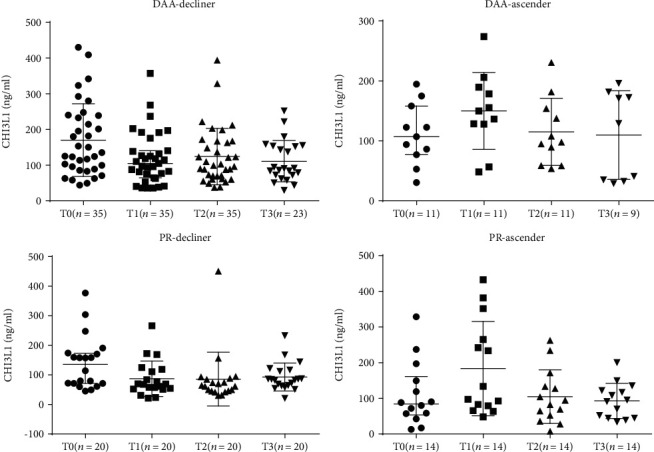
The dynamic changes of serum CHI3L1 of DAA-treated patients and PR-experienced patients in different subgroups. T0: at the baseline; T1: at the end of treatment; T2: at 24 weeks after treatment; T3: at 48 weeks after treatment.

As shown in Table 2, for the DAA-treated group as a whole or the decliner subgroup of the DAA treatment regimens, there was a continued decline in the CHI3L1 level from the baseline to the end of treatment to weeks 24 and 48 after treatment (Table 2 and Figure 5). However, for the PR treatment group, the decline took longer to achieve statistical significance and was not significant until week 24 after treatment (*P* = 0.016). Most of the amplitude of the decrease occurred between T1 and T2, but the decreasing trend was reversed at T3. Thus, the DAA-treated group seemed to exhibit a sustained reduction in fibrosis, whereas the PR-treated group did not.

### 3.7. Dynamic Changes in the CHI3L1 Levels in LC Patients and Non-LC Patients after HCV Eradication

**Figure 6:**
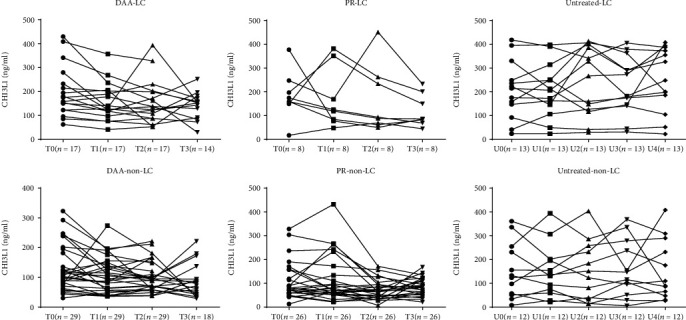
The dynamic changes of CHI3L1 level in LC and non-LC patients.

Cases were classified as non-LC and LC groups for analysis alterations of serum CHI3L1 contents. In the LC group receiving DAA treatments, the level of CHI3L1 was significantly decreased between T0 and T1, continued to decrease at a much slower pace after the end of treatment, and remained significantly decreased at week 24 or week 48 posttreatments (Table 3 and Figure 6). For the noncirrhotic patients, although the CHI3L1 levels at T0 were approximately 40% lower in the non-LC group than in the LC group, similar patterns of decline were observed, suggesting that DAA treatments effectively reduced liver fibrosis regardless of the cirrhosis status before treatment.

For the PR-experienced cirrhotic patients, there was no huge change in the CHI3L1 levels among the follow-up. For the PR-experienced noncirrhotic patients, no obvious alteration was observed immediately after treatment compared with the baseline level, but the difference reached statistical significance at 24 weeks posttreatment (P02 = 0.02, Table 3). However, this reduction was short-lived, and no significant change was observed between the baseline and 48 weeks posttreatment.

For the untreated cirrhotic patients, liver fibrosis measured as the CHI3L1 level increased to 198.26 (264.90) at the week 96 follow-up vs. 158.17 (177.14) at the baseline, which is an approximately 25% increase in approximately 2 years (96 weeks). For the untreated non-LC patients, changes detected in the follow-up for 96 weeks showed no statistical significance.

T0: baseline. T1: at the end of treatment; T2: at 24 weeks after treatment; T3: at 48 weeks after treatment. U0 to U4 indicate the baseline and follow-up at weeks 24, 48, 72, and 96.

## 4. Discussion

Repeated biopsy is the method used to evaluate the worsening of liver fibrosis or monitor the progression of liver fibrosis in many previous studies [3, 15]. However, biopsy may be associated with bleeding or death due to its invasive nature, and being a subjective analysis due to its dependence on human examination of stained liver sections, endures variability among pathologists and hospitals. Therefore, there is an urgent need for a better noninvasive method to monitor the progression of liver fibrosis after SVR in CHC patients.

CHI3L1 is able to activate the Wnt/beta-catenin signal pathway, protein kinase B/AKT, and mitogen-activated protein kinase (MAPK) in macrophage [16]. As a result, CHI3L1 serves as the signaling molecule in the upstream to regulate liver fibrosis, which may be used to be the prognostic factor for liver fibrosis [17]. Wang et al. have demonstrated there were positive and moderate correlations of CHI3L1 with LSM prior to the treatment (*r* = 0.412) as well as following the 78-week treatment (*r* = 0.443) among CHB cases. Besides, the values of collagen proportionate area (CPA) in liver tissues showed a correlation with the CHI3L1 contents before and after treatments, which concluded that CHI3L1 is a useful marker [18]. However, the above results mainly came from the studies of liver diseases related to HBV, and there is a lack of data related to CHC patients.

CHI3L1 was examined to be the marker to monitor liver fibrosis among the CHC cases prior to and following treatments in this study. The serum CHI3L1 contents were found to be moderately positively correlated baseline LSM (*r* = 0.615), and the correlation had statistical significance (*P* < 0.001). In addition, the values of AUCs in discriminating liver cirrhosis was 0.939, which suggested that CHI3L1 can serve as the serum marker to detect fibrosis in a noninvasive way. Our findings conformed to those obtained from Saitou et al.'s study, in which the abilities of type IV collagen (CIV), CHI3L1, hyaluronic acid (HA), and amino-terminal peptide in type III procollagen (PIIIP) were investigated for segregating the distinct histological stages of fibrosis. Their results suggested that YKL-40 showed superiority to additional candidate fibrosis markers in the discrimination of mild fibrosis (F0-F1) from the severe one (F2-F4) (YKL-40, AUC = 0.809; HA, AUC = 0.805) [19]. Other studies were similar to our results, Huang et al. showed that CHI3L1 was superior to other traditional serum markers, including HA, PIIIP, laminin (LN), and CIV, for using identifying advanced liver fibrosis in China. CHI3L1 performed the best among the five markers, with an AUC of 0.99 [11]. Rath et al. found that of all serum markers analyzed, including CHI3L1, HA, LN, CIV, matrix metalloproteinase 9 (MMP-9), tissue inhibitors of metalloproteinases 1 (TIMP-1), tissue inhibitors of metalloproteinases 2 (TIMP-2), and a complex of MMP-9 and TIMP-1, CHI3L1 exhibited the highest diagnostic accuracy in the total patient population as well as in the subgroup of patients with HCV-associated liver disease [20].

We found that the serum CHI3L1 level decreased faster in the DAA-treated patients than in the PR-experienced patients, and it continued to decrease in the DAA-treated patients, but the trend was reversed in the PR-experienced patients, suggesting that the fibrosis regression represented by the reduction in the CHI3L1 level is noteworthy and sustainable in the DAA-treated group but less remarkable and not sustainable in the PR-experienced group. Our data confirmed and reinforced the utility of CHI3L1 to be the marker to monitor liver fibrosis among patients with CHC prior to and following treatments. The difference in treatment efficacies for reducing liver fibrosis between DAAs and PR might be due to the difference in their mechanisms of action. For the PR treatment, the mechanism of action of ribavirin includes suppressing inosine monophosphate dehydrogenase (IMPDH), inhibition of the capping of viral transcripts and viral polymerase, and suppression of cellular and humoral immune responses [21]. Furthermore, ribavirin can act as a lethal mutagenic agent on viral RNA genomes [21]. Interferons have antiproliferative and immunomodulatory properties in light of viral infection, which upregulate antigens in the major histocompatibility complex, cytotoxic T cell and natural killer activities, and production of cytokines and endogenous interferon [22]. In contrast, DAAs target HCV RNA-dependent RNA polymerase (RdRp) to inhibit viral replication [23].

Based on the changes in the CHI3L1 levels, we were able to stratify treatment responses into four categories according to whether their influence on fibrosis is greater or less than 20% (increase or decrease) from the baseline levels. This stratification system might be useful in monitoring the responses of patients to different treatment regimens and in temporal monitoring of fibrosis regression or progression.

The large dynamic ranges of the CHI3L1 level, which are on the order of tens to hundreds, make CHI3L1 more sensitive in detecting smaller changes in fibrosis at shorter intervals (e.g., weeks rather than months or years) than other noninvasive markers that have much smaller dynamic ranges (e.g., within single-digit values for APRI and FIB4 and 2 to 14 or higher for Fibroscan for HCV). This is of great importance for patient management as CHI3L1 can identify those patients who fail treatments earlier. Using CHI3L1, we were able to effectively monitor dynamic changes in fibrosis in time periods as short as 12 weeks or in short intervals of weeks, such as between the baseline and the end of treatment and in later time periods, in HCV patients receiving different treatment regimens. We found that DAA-based treatment regimens were more effective than PR-based treatment regimens: the baseline CHI3L1 level declined continuously immediately after treatment and at weeks 24 and 48 following the treatment; by contrast, the decline in the PR treatment group took longer to achieve statistical significance, which occurred at week 24 after treatment (*P* = 0.016 between T0 and T2), but the decreasing trend was reversed at week 48 (T3) posttreatment.

Finally, dynamic variations of CHI3L1 contents in LC cases, a group of patients who would benefit most from a sensitive and noninvasive test, were analyzed. We found that the baseline CHI3L1 level significantly decreased compared with that immediately after treatment in the LC group receiving DAA treatments and continued to decrease at a much slower pace immediately after treatment, and it was still markedly reduced at week 24 or week 48 posttreatment, suggesting that even for LC patients, CHI3L1 can sensitively detect early and small changes in liver fibrosis after treatment for the LC patients, a task not easily achievable with less sensitive technologies such as FibroScan. Persico's study proved the result, and they demonstrated that LSMs significantly decreased of 749 CHC patients with F3/F4 fibrosis at the end of treatments. Therefore, the regression of liver fibrosis in the F3/F4 patients was notable and has been confirmed [24]. For the untreated cirrhotic patients, liver fibrosis measured as the CHI3L1 level increased approximately 25% in approximately 2 years (96 weeks), while there were no significant changes in the non-LC patients at such intervals. This result indicates that liver fibrosis progresses much more quickly in LC patients than non-LC patients, and timely treatment and monitoring of LC patients is critical to delaying the progression of liver disease.

Our studies have several limitations. The sample size is still small and the follow-up time is still short, and we could not assess the association between improvement in fibrosis measured as the CHI3L1 level after an SVR and later HCC occurrence rates.

In conclusion, we showed that CHI3L1 is a sensitive noninvasive serum marker for monitoring changes in fibrosis during treatments and after achieving an SVR and for assessing the efficacies of HCV treatments. CHI3L1 has the potential to allow the identification of early treatment failure for timely switching to an alternative treatment and to allow monitoring of the progression of fibrosis as a hazard factor for liver cirrhosis and HCC.

## Figures and Tables

**Table 1:** Baseline characteristics.

	DAAs-treated (*n* = 46)	PR-experienced (*n* = 34)	Untreated (*n* = 25)	*P* value^∗^
Age	56.76 ± 14.53	48.12 ± 11.64	58.24 ± 16.60	0.01
Male/female	23/23	23/11	12/13	NS
HCV-RNA log10 (IU/ml)	6.40 (2.42)	6.24 (1.69)	5.72 (1.23)	0.507
HCV genotype (1b/2a)	33/13	24/10	-/-	NS
LC/non-LC	17/29	8/26	14/11	NS
ALT (IU/l)	26 (46)	42 (47.25)	41 (28.50)	0.176
AST (IU/l)	32 (34)	30 (34.75)	46 (45)	0.307
PLT (10^9^/l)	138.8 ± 58.6	149.6 ± 67.1	131.2 ± 66.3	0.563

HCV: hepatitis C virus; ALT: alanine aminotransferase; AST: aspartate aminotransferase; PLT: platelet; NS: no significance. ^∗^DAA-treated vs. PR-experienced vs. untreated.

**Table 2:** Dynamic changes of serum CHI3L1 levels in SOF-based regimens treated patients and PR-experienced patients.

	T0	T1	T2	T3	P01	P12	P23	P02	P03
DAA-all	123.79 (118.55)	118.20 (103.68)	98.46 (96.49)	91.19 (98.63)	0.001	0.474	0.05	0.001	<0.001
DAA-ascender (11/46)	107.13 (81.24)	149.85 (61.72)	97.90 (95.31)	128.97 (143.23)	0.003	0.075	0.678		
DAA-decliner (35/46)	135.79 (150.17)	104.79 (76.053)	99.03 (97.936)	90.69 (81.94)	<0.001	0.682	0.029		
PR-all	89.15 (110.59)	81.01 (112.13)	69.98 (51.44)	85.40 (61.03)	0.555	0.003	0.925	0.016	0.036
PR-ascender (14/34)	84.06 (107.79)	115.50 (210.724)	88.48 (94.6171)	93.18 (80.57)	0.001	0.004	0.272		
PR-decliner (20/34)	135.86 (98.9372)	68.56 (64.06)	63.05 (41.88)	83.90 (52.07)	<0.001	0.263	0.247		

Median and interquartile ranges in brackets are shown. T0: the baseline; T1: the end of treatment; T2: 24 weeks after treatments; T3: 48 weeks after treatments. P01: *P* values comparing T0 to T1; P02: *P* values comparing T0 to T2; P12: *P* values comparing T1 to T2; P23: *P* values comparing T2 to T3.

**(a):** 

	T0	T1	T2	T3	P01	P12	P23	P02	P03
DAAs	123.79 (118.55)	118.20 (103.68)	98.46 (96.49)	91.19 (98.63)	0.001	0.474	0.05	0.001	<0.001
DAA-LC (17/46)	174.91 (133.40)	131.81 (94.4)	136.85 (112.67)	152.94 (84.71)	0.006	0.407	0.397	0.158	0.074
DAA-non-LC (29/46)	107.13 (114.81)	95.76 (89.49)	90.16 (74.45)	75.38 (61.95)	0.02	0.905	0.913	0.014	0.003
PR	89.15 (110.59)	81.01 (112.13)	69.98 (51.44)	85.40 (61.03)	0.555	0.003	0.925	0.016	0.036
PR-LC (8/34)	164.02 (159.33)	139.74 (255.07)	69.59 (165.16)	123.67 (82.50)	0.401	0.263	0.92	0.484	0.05
PR-non-LC (26/34)	79.98 (99.02)	78.12 (70.94)	69.98 (50.21)	82.45 (43.68)	0.218	0.009	0.99	0.02	0.159

**(b):** 

	U0	U1	U2	U3	U4	P01	P04
Untreated	158.17 (177.14)	162.01 (165.33)	160.04 (273.34)	174.02 (189.42)	198.26 (264.90)	0.946	0.15
Untreated-LC (14/25)	194.73 (172.46)	200.99 (131.11)	213.41 (267.43)	180.42 (179.04)	224.18 (267.93)	0.683	0.048
Untreated-non-LC (11/25)	133.08 (204.12)	130.73 (142.23)	151.62 (225.07)	147.23 (227.68)	89.50 (242.97)	0.594	0.89

Treated: T0: baseline; T1: the end of the treatment; T2: 24 weeks after treatments; T3: 48 weeks after treatments. Untreated: U0: baseline; U1: follow-up at week 24; U2: follow-up at week 48; U3: follow-up at week 72; U4: follow-up at week 96. P01, P12, P23, P34, and P04 indicate *P* values for comparing respective time points of T0 to T3 or of U0 to U4.

## Data Availability

All data generated or analyses during this study are included in this published article.

## Abbreviations

CHC: Chronic hepatitis C
WHO: World Health Organization
HCV: Hepatitis C virus
HCC: Hepatocellular carcinoma
DAAs: Direct-acting antivirals
SVR: Sustained virological response
PegIFN/RBV, PR: Pegylated interferon/ribavirin
LF: Liver fibrosis
TE: Transient elastography
CHI3L1: Chitinase 3-like protein 1
HSC: Hepatic stellate cells
SOF: Sofosbuvir
HIV: Human immunodeficiency virus
LSM: Liver stiffness measurement
ELISA: Enzyme-linked immunosorbent assay
SD: Standard deviation
ROC: Receiver operating characteristic
MAPK: Mitogen-activated protein kinase
CPA: Collagen proportionate area
CIV: Type IV collagen
HA: Hyaluronic acid
PIIIP: Type III procollagen
LN: Laminin
MMP-9: Matrix metalloproteinase 9
TIMP-1: Tissue inhibitors of metalloproteinases 1
TIMP-2: Tissue inhibitors of metalloproteinases 2
IMPDH: Inosine monophosphate dehydrogenase
RdRp: RNA-dependent RNA polymerase
EASL: European Association for the Study of the Liver.
